# A bead-based cleavage method for large-scale identification of protease substrates

**DOI:** 10.1038/srep22645

**Published:** 2016-03-03

**Authors:** Chunli Wang, Mingliang Ye, Xiaoluan Wei, Yangyang Bian, Kai Cheng, Hanfa Zou

**Affiliations:** 1 Key Laboratory of Separation Sciences for Analytical Chemistry, National Chromatographic R&A Center, Dalian Institute of Chemical Physics, Chinese Academy of Sciences, Dalian 116023, China; 2 Institute of Cancer Stem Cell, Dalian Medical University, Dalian 116044, China

## Abstract

Proteolysis is a major form of post translational modification which occurs when a protease cleaves peptide bonds in a target protein to modify its activity. Tracking protease substrates is indispensable for understanding its cellular functions. However, it is difficult to directly identify protease substrates because the end products of proteolysis, the cleaved protein fragments, must be identified among the pool of cellular proteins. Here we present a bead-based cleavage approach using immobilized proteome as the screening library to identify protease substrates. This method enables efficient separation of proteolyzed proteins from background protein mixture. Using caspase-3 as the model protease, we have identified 1159 high confident substrates, among which, strikingly, 43.9% of substrates undergo degradation during apoptosis. The huge number of substrates and positive support of *in vivo* evidence indicate that the BBC method is a powerful tool for protease substrates identification.

Proteolytic processing, as an indispensable post translational modification (PTM), regulates many important cellular processes including apoptosis, immune function, tumorigenesis, invasion and metastasis^1^,^2^,^3^. It occurs when a protease cleaves one or more peptide bonds in a target protein to modify its activity. Altered protease expression or activation contributes to aberrant proteolysis of substrates and eventually to disease progression. Thus the proteases are considered as the promising drug targets^4^,^5^. Proteins/peptides with PTMs, like phosphorylation and glycosylation, could be enriched by affinity chromatography because additional chemical groups are covalently coupled onto the side chains of the proteins. However, the end products of proteolysis reaction, the cleaved proteins have no such groups to allow selective enrichment and thus it is difficult to directly identify protease substrates especially among the pool of cellular proteins^6^,^7^.

Benefit from the rapid development of proteomics techniques, two types of high-throughput methods have been developed for identification of protease substrates^6^,^7^,^8^. The first type is the gel-based methods that combine gel electrophoresis with quantitative proteomics to identify a large number of caspase substrates from proteolytic peptide fragments^9^,^10^,^11^,^12^,^13^. The Cravatt lab has developed the PROTOMAP method for screening of proteolyzed proteins in staurosporine-induced apoptosis process^12^. In this method, lysates from apoptotic cells and control cells are first separated by SDS-PAGE in adjacent lanes and the proteolyzed proteins are identified by comparing the altered migration patterns and intensities between the two samples. The significant feature of this method is that it enables direct visualization of the topography and magnitude of proteolytic events on a global scale. However, considering the fact that proteolyzed proteins present in the huge number of background proteins, this method is preferable to identify high abundant substrates. The second type is N-terminal peptide labeling methods which mainly rely on negative or positive enrichment of cleaved N-terminal peptides^14^,^15^,^16^,^17^,^18^,^19^,^20^,^21^. The Wells lab has developed a positive enrichment method using an engineered enzyme termed subtiligase to selectively biotinylate free N-termini of protein fragments from etoposide-induced apoptosis process^16^. This method allows selective enrichment of N-terminal peptides of proteolyzed proteins from the tremendously noisy protein background, thus has the potential to identify lower abundant substrates. A negative method of terminal amine isotopic labeling of substrates (TAILS) has been developed by the Overall lab by using dendritic polyglycerol aldehyde polymers to remove tryptic and C-terminal peptides^19^. This method achieves broad protease substrate coverage and identifies post-translationally modified N-termini sites. However, because each substrate is identified by single-terminal peptides and proteins with terminal peptides not readily identifiable via MS are excluded, the N-terminal peptide labeling methods have their limitation in substrates identification.

In this study, we propose a new type of method, bead-based cleavage (BBC) method using immobilized proteome as the screening library, for large-scale identification of *in vitro* protease substrates. This method enables efficient separation of proteolyzed proteins from background protein mixture. Using caspase-3 as the model protease, we have identified 1159 high confident substrates, among which, strikingly, 43.9% of substrates undergo degradation during apoptosis. The huge number of substrates and positive support of *in vivo* evidence indicate that the BBC method is a powerful tool for protease substrates identification.

## Results

### Workflow for Bead-Based Cleavage (BBC) Method

**Figure 1:**
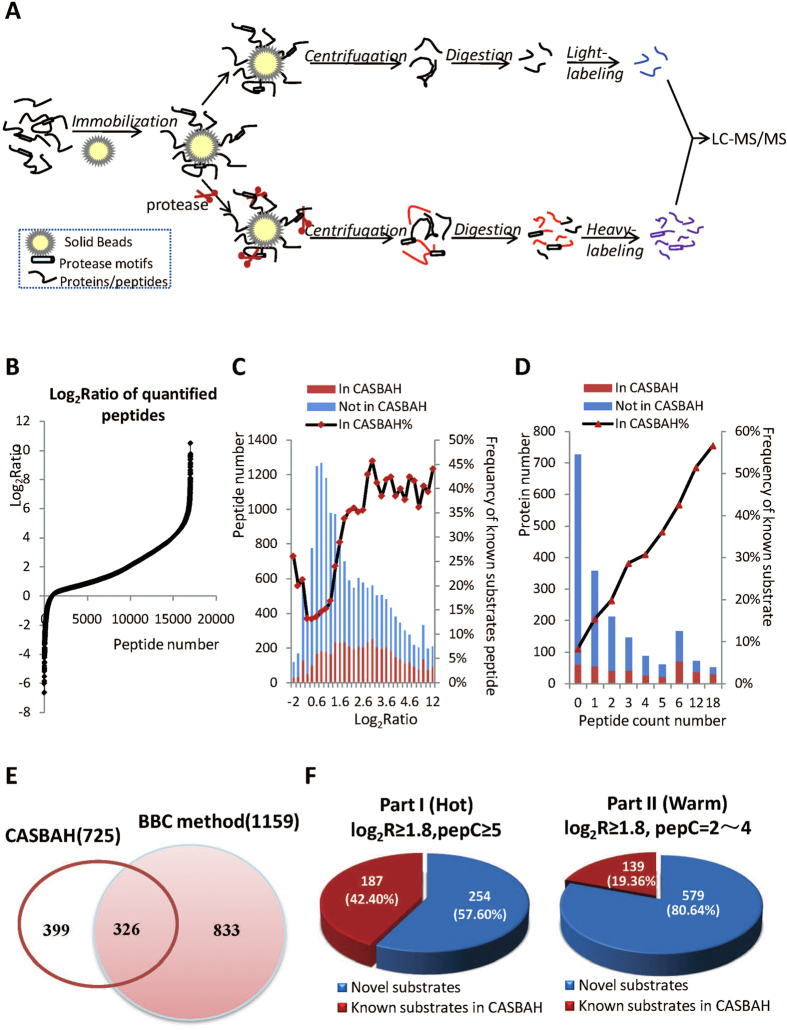
Screening of protease substrates by BBC method. (**A**) Workflow of the bead-based cleavage (BBC) method for high-throughput screening of protease substrates. (**B**) Log_2_Ratio distributions of all quantified peptides (H/L = caspase-3 treated/untreated). (**C**) The percentages of peptides derived from substrate proteins listed in CASBAH database for different log2Ratio values. (**D**) The dependence of the percentages of peptides derived from substrate proteins in CASBAH database on the peptide count numbers with log_2_Ratio ≥ 1.8. (**E**) Overlap between substrates identified by BBC method and known substrates in CASBAH database. (**F**) Percentage of known substrates for identified hot and warm substrates.

To sensitively identify protease substrates, it is crucial to separate the proteolyzed proteins from other cellular proteins. In this study, we propose a bead-based cleavage (BBC) method for large-scale identification of *in vitro* protease substrates. This method enables efficient separation of proteolyzed proteins from background protein mixture. The workflow is shown in Fig. 1A. Proteins in cell lysate are firstly covalently immobilized onto Agrose beads, and then immobilized proteome is incubated with the active protease. Theoretically, only protease substrates could be cleaved and the resulted protein fragments are released into the free solution from beads. However, due to the presence of trace active endogenous proteases in the immobilized proteome on the beads, protein fragments from proteins other than protease substrates may be generated as well. Therefore, a control experiment without addition of protease is also performed in parallel and quantitative proteomics technique is applied to differentiate the substrates from the background degraded proteins. As shown in Fig. 1A, the protein fragments from the two groups are further digested by trypsin to make peptides more suitable for identification by MS. After that, the resulted peptides from protease treated sample and untreated sample are separately labeled with heavy and light stable isotopic tags. LC-MS/MS is then applied to analyze the pooled sample. The substrates are finally determined based on the quantified results.

### Screening of Caspase Substrates by BBC Method

We used the apoptosis executioner caspase-3 as the model protease and Jurkat cell proteome as the screening library to evaluate the performance of BBC method. The proteins in the cell lysate from Jurkat cells were immobilized onto beads. The *in-vitro* digestion and dimethyl labeling procedures were performed as that described in the workflow. Three replicate 2D LC-MS/MS analysis of dimethyl labeled peptide mixture from caspase-3 treated and untreated samples resulted in the quantification of 16994 peptides and 2814 proteins (Supplementary Table S1). As expected, majority of peptides had higher ratios in the caspase-3 treated sample (Fig. 1B). However, there were still unexpected peptides degraded due to the trace endogenous proteases on beads. Therefore, the quantified ratios could be used to differentiate the peptides of background proteins from those by caspase catalyzed cleavages. The potential substrates were screened according to the ratios of up-regulated peptides. Usually, log_2_Ratio > 1 is considered as an acceptable standard for up-regulated peptides. In our results, over one third (43.02%) of peptides were up-regulated. Clearly the incubation of the immobilized proteome with caspase released many protein fragments.

In degradomics, for proteases with a significant number of substrates are known, “cutoffs” can be determined based upon the ratios for known substrates^3^. We then determine more appropriate filtering criteria to differentiate the substrates from the background proteins. A comprehensive list of the caspase substrates (725) identified by numerous studies are archived in the database of CASBAH^22^ (Supplementary Table S2). This database helps us to obtain the best filtering criteria. We first investigated the percentages of peptides derived from substrate proteins included in CASBAH for different quantified ratios (Fig. 1C). A general trend was observed that the higher the ratio the bigger the percentage of peptides from known substrates. When the log_2_Ratio increased from 1.0 to 1.8, the percentages of peptides from known substrates strikingly increased from 15.2% to 33.8%. When the log_2_Ratio was over 1.8, the percentage of peptides from known substrates was all over 33.0%. In total, 1886 proteins were identified by peptides with log_2_Ratio ≥ 1.8. Among these proteins, 386(20.5%) were known substrates in CASBAH. However, with further increase of the peptide ratio, the percentage of peptides from known caspase substrates only slightly increased, indicating the insufficient differential power of peptide ratio alone.

It is well known that the proteins identified with multiple peptides are more confident. We expected that the number of quantified peptides could be an additional criterion for confident substrate identifications. The dependence of the percentage of known substrates on the peptide count numbers with log_2_Ratio ≥ 1.8 was shown in Fig. 1D. It was observed that the more number of peptides with log_2_Ratio ≥ 1.8, the higher percentage of known substrates. There were 1159 proteins identified by at least 2 peptides with log_2_Ratio ≥ 1.8. Among these proteins, 326 proteins (28.1%) were known substrates (Fig. 1E). The percentage was obviously higher than that obtained by using peptide ratio alone as the criterion. Clearly the number of quantified peptides with log_2_Ratio ≥ 1.8 was an effective criterion to filter out the background cleavage events. Therefore, in this study we set log_2_Ratio ≥ 1.8 and peptide count number ≥2 as the final optimized criteria. After filtering our quantified dataset, 1159 putative caspase substrates were screened (Supplementary Table S3). Comparing with the conventional cutoff (log_2_Ratio > 1 of peptide considered as the up-regulated peptides), the criteria here used could help us to get more specific substrates. The substrates identified by at least 5 peptides, named as “hot substrates”, are of high confidence, however these proteins are more likely to be high abundant proteins and have been well explored which is confirmed by the fact that over 40.0% of these proteins are known caspase substrates (Fig. 1F). To discover new *bona fide* substrates, the low abundant putative substrates identified by 2–4 peptides named as “warm substrates” may have more chance. The high percentage of known substrates and the huge number of identified substrates indicate the excellent performance of this BBC strategy.

**Figure 2:**
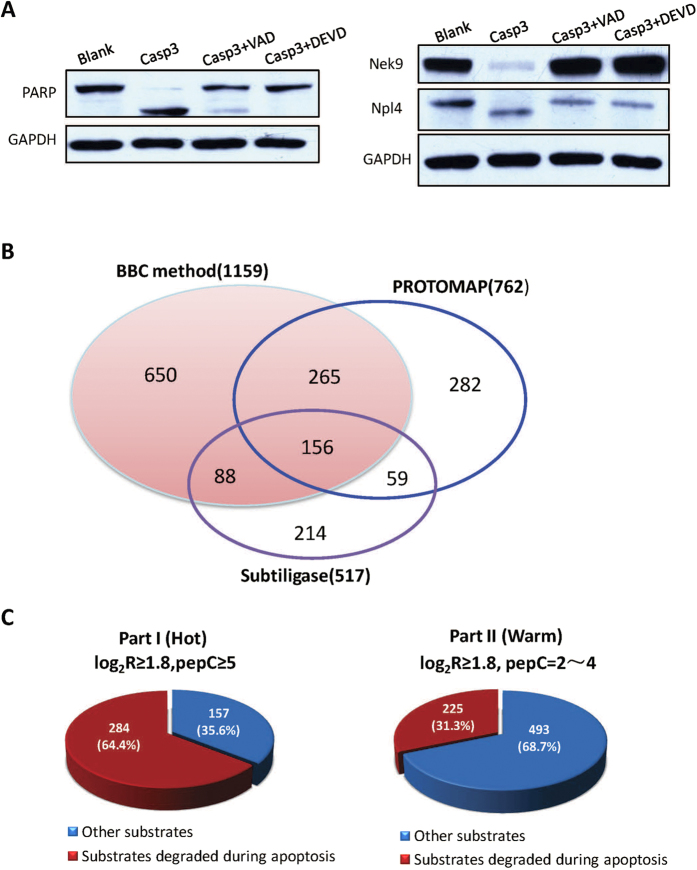
Substrates degraded during apoptosis. (**A**) Western blot analysis of *in vitro* cleavage of PARP, Nek9, Npl4 by caspase-3 with/without inhibitors. PARP as the known substrate of caspase-3 was used as a positive control, and GAPDH was used as the internal control. (**B**) Comparison of *in vitro* substrates identified by BBC method with degraded proteins during apoptosis identified by PROTOMAP and Subtiligase methods; (**C**) Percentage of substrates in “Hot” and “Warm” dataset found to be degraded during apoptosis identified by PROTOMAP and Subtiligase methods.

Above substrates were identified by using the immobilized proteome as the screening library. We then applied conventional in-solution based approach to test if the identified substrate proteins could be cleaved *in vitro* by caspase-3. Nek9 is a NIMA-family kinase required for normal mitotic progression and spindle organization. Dysfunction of Nek9 is involved in cancer progression^23^,^24^. In our study, Nek9 was identified by 6 up-regulated peptides. We tested if this protein could be cleaved in solution by caspase-3, and whether this degradation could be rescued by inhibitors of caspase-3. The cell lysate was incubated with or without caspase-3, while the rescue experiment used the inhibitors, Ac-DEVD-fmk or Ac-VAD-fmk in the presence of caspase-3. Poly [ADP-ribose] polymerase-1 (PARP-1), a well-known substrate of caspase-3^10^,^25^, was used as a positive control (Fig. 2A). As Fig. 2A shown, the band of Nek9 faded out after caspase-3 added, rescued in the presence of the two inhibitors, confirming that Nek9 were degraded by caspase-3. Npl4, binding with p97 and UFD1L, regulates G2/M checkpoint signaling and NF-κB activation^26^,^27^. However, it is unknown whether Npl4 involves in caspase-3-mediated apoptosis process. Here, for the first time we figured out that Npl4 was a novel substrate of caspase-3, as Fig. 2A shown. The above two *in vitro* verification experiments showed that caspae-3 substrates identified by BBC method could also be cleaved when free cell lysate was used as the library.

### Characterization of Cleavage Sites for the Identified Substrates

As one of post-translational modifications, cleavage of substrates by protease produces protein fragments. To make the protein fragments that released from the beads by caspase-3 more suitable for MS analysis, trypsin is used to cut the protein fragments into small peptides. Thus the identified and quantified peptides could be generated due to the cleavage by either capase-3 or trypsin or both of them. In this study, we quantified 7086 peptides with log_2_Ratio ≥ 1.8 from the 1159 putative substrates. Except terminal peptides, two cleavage sites could be read from the identified peptides. Those peptides resulted in the identification of 11897 unique cleavage sites. As expected, majority of the cleavage sites (89.6%) were generated by trypsin and minority of cleavage sites (10.4%) were generated by caspase-3 and other proteases. This hinted that protein fragments releases by caspase-3 were so long that it was necessary to perform second digestion. As the cysteine-aspartic acid protease, caspase-3 has the preference for cleaving motif DEVD, DELD, DDXD and DQXD^28^,^29^. In our results, there were 721 unique D cleavage sites identified, among which the DXXD motifs were 29.8% including 30 DEXD motifs, 27 DLXD motifs and 23 DDXD motifs (Supplementary Table S4). All the motifs screened by the BBC method and the known caspase-3 cleavage motifs^29^ were respectively uploaded to WebLogo for analysis^30^ (Supplementary Fig. S1A). The consistent specificity with known dataset further illustrates the reliability of the BBC method.

Among 1159 putative substrates, 485 (41.8%) substrates were identified with at least one D cleavage site. And 64 D sites from 60 substrates were consistent with those included in CASBAH. For example, Serine/threonine-protein kinase (PAK2) was identified as a hot substrate by 7 up-regulated peptides (Supplementary Fig. S1B). Cleavage sites D148 (PPEKD.GFPSGTPALNAK.GTEAP) and D212 (SIYTR.SVIDPVPAPVGDSHVD.GAAKS) were up-regulated by 5.85-fold and 35.85-fold respectively, revealing them to be cleaved by caspase-3. Proteolytic cleavage of C-terminal D212 site removed most of the regulatory domain, generating a constitutively active PAK-2p34 catalytic fragment which was involved in apoptosis. And it was reported that caspase activation stimulated by apoptotic stimuli appeared to turn the anti-apoptotic activity of PAK2 into a pro-apoptotic activity of PAK2-p34^31^,^32^,^33^. Another protein, breast carcinoma-amplified sequence 2 (BCAS2) was newly revealed to negatively regulate P53. Reducing the expression of BCAS2 increased p53-dependent apoptosis in cancer cells^34^. In this study, BCAS2 (warm substrate) was identified by two up-regulated peptides and the cleavage site D14 (EVVVD.ALPYFDQGYEAPGVR.EAAAA) was up-regulated by 10.18-fold (Supplementary Fig. S1B). Recently, the Wells’s team also discovered that the D148 site of PAK2 and D14 site of BCAS2 were cleaved during staurosporine-induced apoptosis process^16^. Therefore, caspase-3 is likely to be the key protease for cleaving these sites. The above cleavage sites consistent with known data also indicate the reliability of the BBC method.

### Significant Fraction of Identified Substrates Undergo Degradation during Apoptosis

Caspase-3 is the main executive enzyme of apoptosis. The *bona fide* caspase-3 substrates should be cut during apoptosis under some stimuli. To confirm whether several hundreds of proteins are degraded during apoptosis by western blot is extremely labor-intensive. Fortunately, two research teams have identified hundreds of degraded proteins by using two different large scale approaches during Jurkat cell apoptosis. The Cravatt’s team has observed 762 degraded proteins with 169 cleavage sites by using PROTOMAP method (combined results from two papers published^11^,^12^) and the Wells’s team has observed 517 degraded proteins with 641 cleavage sites by using Subtiligase method (also combined results from two papers published^16^,^21^). Totally 1064 proteins have been found to undergo degradation during Jurkat cell apoptosis (Supplementary Table S5). These *in-vivo* datasets offer us a high throughput way to validate the substrates identified in this study. We compared the 1159 substrate proteins identified by the BBC method with the 1064 proteins degraded during apoptosis. To our surprise, there were 509 substrates in common between these two datasets. As Fig. 2B shown, the overlap ratio (29.7%) was even higher than that between the substrates identified by the two *in vivo* studies (20.2%). Strikingly, 64.4% (284) were cut during apoptosis among the 441 hot substrates while 31.3% (225) were cut among the warm substrates (Fig. 2C). Bring together, 509 (43.9%) substrates identified by BBC method were observed to be degraded during apoptosis. The high percentage of overlapping indicated that the possible conformation change and steric hindrance resulted from protein immobilization did not contribute too much false positive identification. The 509 overlapped substrate proteins were of high confidence because they were observed not only to be cut by caspase-3 *in vitro* but also to be degraded during apoptosis. To explore which biological process caspase-3 involved in, we loaded all the 509 high confident substrates to Panther (http://www.pantherdb.org/) for statistical overrepresentation analysis^35^. The whole proteome on beads (4609 proteins) were used as the reference list (Supplementary Table S6). As expected, the 509 high confident substrates were found to be enriched in RNA/mRNA splicing and mRNA metabolic processes, which was consistent with result reported^18^,^21^. However, caspase-3 seemed not to cleave the proteins involved in lipid and carbohydrate metabolic process, and it would be interesting to study the crosstalk between apoptosis and metabolic processes in future (Supplementary Table S7).

## Discussion

Protease substrate discovery experiments can be divided into two broad categories with regard to the biological question they address^6^. Forward (or *in vivo*) experiments address what substrates are cleaved during a biologically relevant event, while reverse (or *in vitro*) experiments address what substrates a particular protease is capable of cleaving. The BBC method presented in this study is best fitted for reverse experiments, which allows the identification of *in vitro* protease substrates in large scale. By using caspase-3 as the model protease and Jurkat cell proteome as the screening libarary, we have succesfully identified 1159 substrate proteins. This is obviously the largest dataset of caspase substrates thus far. Excitingly, over 40% of these substrate proteins (509 out of 1159) were found to undergo degradation, and among them two third of the hot substrates (identified by at least 5 peptides with log_2_Ratio ≥ 1.8) were degraded during Jurkat cell apoptosis. To the best of our knowledge, above fractions are highest in the coverage of known substrates for reverse experiments. The huge number of identified susbtrates and the positive support of *in vivo* evidence indicate the BBC method presented in this study is a sensitive and reliable approach for large-scale identification of *in vitro* protease susbtrates.

In principle, the reported large-sclae protease substrate discovery methods, either the gel-based methods or the N-terminal peptide labeling methods, could be used for reverse (or *in vitro*) experiments. The gel-based methods are known to bias to high abundant proteins while the N-terminal peptide labeling methods exclude the identification of substrates with their terminal peptides not readily detected by MS. The BBC method identifies *in vitro* substrates in a less biased way. The immobilized proteome is used as the screening library. The substrate proteins are released from the beads, which effectively eliminates the interference of huge amount of background proteins presented in the cell lysate and thus obtains high sensitivity for the identification of protease substrates. Comparing with the conventional reverse (or *in vitro*) experiments where free cell lysate is incubated with protease of interest, this BBC method allows direct exchange of buffers as all proteins are immobilized onto beads. This feature makes the *in vitro* cleavage assay to be performed in detergent-free buffer where proteins are folded in their native conformation. This could be the main reason why the *in vitro* substrates identified by this method have higher probability to be genuine *in vivo* substrates. Considering that tracking protease substrates is indispensable for understanding whole communities of proteases and their application as new biomarkers and drug targets, the BBC method presented in this study could be a powerful tool for the fast and reliable large-scale screening of protease substrates that will help us monitor and treat cancer.

One advantage of the N-terminal peptide labeling methods is that they are able to identify cleavage sites efficiently because the N-terminal peptides derived from the cleaved protein fragments are selectively enriched and analyzed^14^,^15^,^16^,^17^,^18^,^19^,^20^,^21^. However, this is not the case for the BBC method and the gel-based method. In the BBC method, the protein fragments with site information are firstly released from the beads into solution and then subjected to further digestion by trypsin, which make the tryptic peptides overwhelm the peptides fragments carrying the caspase cleavage sites. As a result, the N- or C-terminal peptides are less efficiently identified due to the shotgun nature of proteomics analysis. This is also the case for the gel based methods where the separated protein fragments are further digested by trypsin for protein identification. In our results only 485 (41.8%) putative substrates were identified with cleavage sites. While in the gel based method, like PROTOMAP method, only 136 (17.8%) substrates containing cleavage sites are observed^11^,^12^. Therefore, both the BBC method and the gel-based method cannot localize all cleavage sites on the substrate. Clearly further improvement in this aspect should be performed in the future.

## Methods

### Cell Lines and Reagents

Human Jurkat T cell line (TCHU123, ATCC) was purchased from Cell Bank of Chinese Academy of Sciences (Shanghai, China). RPMI 1640 medium, penicillin and streptomycin were obtained from Gibco Invitrogen Corporation (Carlsbad, CA). Fetal bovine serum was obtained from Biochrom AG (Berlin, Germany). 2-[4-(2-Hydroxyethyl)-1-piperazinyl]ethanesulfonic acid(HEPES), Sodium chloride (NaCl), ethylenediaminetetraacetic acid (EDTA), dithiothreitol (DTT), iodoacetamide (IAA), trifluoroacetic acid (TFA), formic acid (FA), protease inhibitor cocktail, alkaline phosphatase (AP), sodium cyanoborohydride (NaBH_3_CN), formaldehyde solution (CH_2_O, 37%) and formaldehyde-d2 solution (CD_2_O, 20%) were obtained from Sigma-Aldrich (St. Louis, MO). Phenylmethylsulfonyl fluoride (PMSF) was purchased from AMERESCO (Solon, Ohio, USA). Sequencing trypsin and phosphatase inhibitor cocktail tablets (PhosSTOP) were purchased from Roche (Mannheim, Germany). Acetonitrile (ACN) and 25% ammonia solution (NH_3_·H_2_O) were purchased from Merck (Darmstadt, Germany). HLB C18 cartridges were provided by Waters (Milford, MA). Glycine and Triton X-100 were from Bio Basic Inc (Amherst, NY, USA). Caspase-3 was obtained from BD Bioscience (Franklin Lakes, NJ, USA). z-DEVD-fmk and z-VAD-fmk were from MBL (Nagoya, Japan). Leupeptin and aprotinin were from Bachem (Torrance, CA, USA) and Roche (Mannheim, Germany), separately. CNBr-activated Sepharose™ 4B was obtained from GE Healthcare (Pittsburgh, PA, USA). All the water used in the experiments was prepared using a Milli-Q system (Millipore, Bedford, MA). All the chemicals were of analytical grade except acetonitrile, which was of HPLC grade.

### Cell Cultures and Protein Extraction

Human Jurkat T cell line were grown at 37 °C under 5% CO_2_ in RPMI 1640 media containing 10% fetal bovine serum, 100 unit/mL penicillin and 100 μg/mL streptomycin. About 10^8^ cells suspensions were centrifuged at 1000 g and the pellets were washed with ice-cold PBS for three times. Cells were resuspended in an ice-cold non-denaturing lysis buffer containing 1 mM DTT, 1 mM EDTA, 1% (v/v) Triton X-100, 1 mM PMSF (added freshly), 2% (v/v) protease inhibitor cocktail (added freshly) and phosphatase inhibitor cocktail (1 tablet/10 mL lysis buffer, added freshly) in 50 mM HEPES (pH = 7.4), and ultrasonicated in an ice-bath. The lysate was centrifuged at 16000 g for 20 min at 4 °C, and the supernatant was collected. The protein concentration in supernatant was determined to be 6.5 mg/mL by BCA method. The protein lysate was stored at −80 °C for further analysis.

### Protein Immobilization

Jurkat proteins were immobilized on CNBr-activated sepharose 4B beads according to the manufacturer’s instructions with minor modification. Briefly, 100 mg CNBr-activated sepharose beads were pre-washed by 1 mM HCl and 50 mM HEPES (pH = 7.4) for 3 times in turn. Then 0.7 mL protein lysate was captured on the pre-washed CNBr-activated sepharose beads at 4 °C overnight with gently shaking. After immobilization, the beads were washed with a solution containing 4 μM leupeptin, 0.1 μM aprotinin and 1% Triton X-100 in 50 mM HEPES (pH = 7.4) to remove the non-specific absorbed proteins for three times. Then the excess active groups on beads were blocked by a blocking solution containing 1 M glycine, 4 μM leupeptin, 0.1 μM aprotinin, 2% (v/v) protease inhibitor cocktail and 1%(v/v) Triton X-100 in 50 mM HEPES(pH = 7.4) at 4 °C overnight. After that 50 mM HEPES (pH = 7.4) was used to wash the excess reagents for three times. To determine the amount of proteins immobilized onto beads, we quantified the proteins in lysate before and after the immobilization process by BCA method as we described^36^. The amount of proteins captured onto the beads was determined to be 60–70%.

### *In Vitro* Caspase-3 Cleavage Experiments and Dimethyl Labeling Reaction

The beads were incubated with a solution containing 4 μM leupeptin, 0.1 μM aprotinin and 1 mM DTT in 50 mM HEPES (pH = 7.4) for 0.5 h for further inhibiting endogenous protease activity. Then 50 mM HEPES was used to wash the inhibitors quickly. The beads with captured proteins were divided into two aliquots for *in vitro* cleavage experiments. One aliquot was used in the experimental group where caspase-3 was added for *in vitro* cleavage while the other aliquot was used for the control group without addition of caspase-3. For the both experiments the samples were resuspended in 300 μL proteolytic buffer containing 100 mM NaCl, 1 mM EDTA, 5 mM DTT and 1% (v/v) sucrose in 50 mM HEPES (pH = 7.4). After 5 μg caspase-3 was added to the experimental group while no caspase-3 was added into the control group, the cleavage reaction was carried out at 37 °C for 2 h. After the reaction, the cleaved protein fragments were released into the solution from the beads. The supernatants including caspase-3 substrate peptides and background degrade peptides in both samples were separately collected by centrifugation at 1000 g. Then 100 μL of 25 mM HEPES (pH = 7.4) was used to wash the beads for twice and the supernatants were mixed together, separately. To terminate the cleavage reaction, 2.5 μL of 1 mM caspase-3 inhibitor z-DEVD-fmk was added into the supernatant of the two samples. As the caspase-3 cleaved peptides were too long to be identified by mass spectrometry, second digestion was performed. The supernatants were lyophilized for further trypsin digestion. The cleaved peptides in both samples were resuspended in 100 μL of solution containing 8 M urea in 100 mM TEAB (pH = 8.0), and reduced by 20 mM DTT at 37 °C for 2 h and oxidized by 40 mM IAA at 25 °C for 45 min in dark. Then both of the peptide mixture were diluted to 600 μL with 100 mM TEAB buffer (pH = 8.0). After adding 15 μg sequencing trypsin, the peptide mixture was separately digested at 37 °C overnight.

Stable isotope dimethyl labeling method was used to compare the cleaved peptides between the control and experimental groups. The labeling procedure was followed the on-column stable isotope dimethly labeling method^37^. The control sample was light-labeled while the caspase-3 cleaved peptides were heavy-labeled by using different labeling reagents. Briefly, peptides from two samples were firstly acidic by TFA and loaded onto 30 mg pre-activated HLB C18 column to be desalted. Then the caspase-3 cleaved peptides on the C18 column were labeled with 8 mL of solution containing 4% CD_2_O and 0.6 M NaBH_3_CN in PBS (pH = 7.5) while the control peptides on the C18 column were labeled with 8 mL of solution containing 4% CH_2_O and 0.6 M NaBH_3_CN in PBS (pH = 7.5) for about 1 h. After completely labeled, the light-labeled peptides and heavy-labeled peptides were separately eluted by 2 mL solution (80% ACN, 0.1% TFA) and pooled together to be lyophilized for MS analysis.

For whole proteome analysis, we performed the trypsin digestion of protein immobilized on beads. After 0.35 mL of protein lysate was immobilized on CNBr-activated sepharose 4B beads, the proteins on beads were resuspended in 100 μL of solution containing 8 M Urea in 100 mM NH_4_HCO_3_ (pH = 8.0), and reduced by 20 mM DTT at 37 °C for 2 h and oxidized by 40 mM IAA at 25 °C for 45 min in dark. Then 500 μL of 100 mM NH_4_HCO_3_ (pH = 8.0) were added to dilute the solution. After 15 μg sequencing trypsin was added, the reaction was performed at 37 °C overnight. The digestion of whole protein on beads was desalted by 30 mg C18 column and lyophilized for MS analysis.

### Mass Spectrometry Analysis

Peptides were analyzed by LTQ-Orbitrap XL mass spectrometer (Thermo, San Jose, CA) with SCX-RP separating system in the positive ion mode. The LTQ-Orbitrap XL mass spectrometer was operated in a data-dependent MS/MS acquisition mode. Full mass scan performed in the Orbitrap analyzer was acquired from m/z 400 to 2000 (R = 60000 at m/z 400). The 10 most intense ions from the full scan were selected for fragmentation via collision induced dissociation (CID) in the LTQ. The dynamic exclusion function was set as follows: repeat count 2, repeat duration 30 s, and exclusion duration of 60 s. The dimethyl-labeled peptides were analyzed by two-dimensional SCX-RP separation system for three replicates. Firstly, 1/5 dimethyl-labeled peptides was loaded onto the SCX column, and a series of stepwise elution with salt concentrations of 50, 100, 150, 200, 250, 300, 350, 400, 500 mM and 1000 mM NH_4_Ac (pH = 2.7) were used to elute peptides from SCX monolithic column to the second dimensional C18 separation column. The analysis time of multidimensional separation was about 28 h. Each salt step lasted 10 min followed by 15 min equilibrium with 0.1% FA/H_2_O. Finally, a 140 min RP gradient elution was performed as follows. 0.1% FA in water and 0.1% FA in acetonitrile were used as mobile phases A and B, respectively, and the flow rate was adjusted to 200 nL/min after splitting. The gradient elution was performed with a gradient of 0–3% B in 2 min, 3–25% B in 90 min, 25–35% B in 10 min, 35–80% B in 3 min, and 80–100% B in 2 min, and 100% B in 30 min. For whole proteome analysis, 1/10 tryptic peptides were loaded onto the above SCX-RP system for two-dimensional analysis and two replicates were performed.

### Database Searching and Quantitative Analysis

Raw MS/MS spectra were searched using MaxQuant (V1.3.0.5) against the Swiss-Prot human database (20131211downloaded) with 1% false discovery rate (FDR), allowing for precursor-ion mass tolerance, 10 ppm; fragment-ion mass tolerance, 0.5 Da. For labeled peptides, the parameters were set as follows: enzyme, semi-trypsin; missed cleavage, 2; static modification, Cys (+57.0215 Da); variable modifications methionine oxidation (+15.9994 Da), lysine and N termini in light dimethylation (+28.0532 Da) and heavy dimethylation (+32.0778 Da). For whole proteome peptide identifications, the parameters were set as follows: enzyme, trypsin (KR/P); missed cleavage, 2; static modification, Cys (+57.0215 Da); variable modifications methionine oxidation (+15.9994 Da).

### Verification of Substrates by Western Blotting

Cells were lysed in an ice-cold lysis buffer containing 1 mM DTT, 150 mM NaCl, 1 mM EDTA, 1% (v/v) Triton X-100, 1 mM PMSF (added freshly), 2% (v/v) protease inhibitor cocktail (added freshly) and phosphatase inhibitor cocktail (1 tablet/10 mL lysis buffer, added freshly) in 50 mM HEPES (pH = 7.4). The protein concentration was determined by BCA method and normalized to a concentration of approximately 2.5 mg/mL. About 1 mg of protein was desalted by spin-6 column for further analysis. The in-solution cleavage reaction was carried out as follows: One was used as a negative control with no caspase-3 added; the second was used for caspase-3 cleavage reaction; either z-DEVD-fmk or z-VAD-fmk was added into the other two groups in the presence of caspase-3 for rescuing experiments. All the four desalted protein lysates were incubated at 37 °C for 2 h. The cleavage reaction was terminated by 6 × loading buffer and boiled at 95 °C for 5-10 min. And all the above samples were separated on 8% SDS-PAGE gel followed by transferred to nitrocellulose membrane. Membranes were blocked at room temperature for 1 h with 5% non-fat dry milk in TBST buffer containing 10 mM Tris-HCl (pH = 7.5), 150 mM NaCl and 0.05% Tween-20. Blots were hybridized with the following antibodies: PARP (1/1000 dilution), Nek9 (1/250 dilution), Npl4 (1/750 dilution) overnight at 4 °C and reacted with secondary antibody at the room temperature for 1 h. Enhanced chemiluminescence reagent was used for detection. GAPDH (1/1000 dilution) was used as the loading and internal control.

## Additional Information

**How to cite this article**: Wang, C. *et al.* A bead-based cleavage method for large-scale identification of protease substrates. *Sci. Rep.* **6**, 22645; doi: 10.1038/srep22645 (2016).

## Supplementary Material

Supplementary Information

Supplementary Table S1

Supplementary Table S2

Supplementary Table S3

Supplementary Table S4

Supplementary Table S5

Supplementary Table S6

Supplementary Table S7
