# Supplementary material for: A bead-based cleavage method for large-scale identification of protease substrates
Source: Sci Rep. 2016 Mar 3;6:22645. doi: 10.1038/srep22645 (PMC4776233; doi:10.1038/srep22645)
Supplement: Supplementary Information [file srep22645-s1.pdf]

Supporting Information

A bead-based cleavage method for large-scale identification of protease substrates

Chunli Wang[1,2], Mingliang Ye[1*], Xiaoluan Wei[1], Yangyang Bian[1], Kai Cheng[1], Hanfa Zou[1*]

1 Key Laboratory of Separation Sciences for Analytical Chemistry, National Chromatographic R&A Center, Dalian Institute of Chemical Physics, Chinese Academy of Sciences, Dalian 116023, China; 2 Institute of Cancer Stem Cell, Dalian Medical University, Dalian 116044, China

*To whom correspondence should be addressed:

Prof. Dr. HanfaZou hanfazou@dicp.ac.cn; Prof. Dr. Mingliang Ye mingliang@dicp.ac.cn

Figure S1. Analysis of caspase-3 specificity. (A) WebLogo of the consensus motif from known caspase-3 substrates and putative substrates identified by BBC method. (B) Peptides identified from substrates PAK2 and BCAS2 by LC-MS/MS in the BBC method.

Table S1. List of all the peptide quantified by LC-MS/MS (Separate File).

Table S2. List of caspase substrate database CASBAH (Separate File).

Table S3. List of putative caspase-3 substrates with peptide count number (Separate File).

Table S4. List of known caspase-3 cleavage sites and caspase-3 specific cleavage sites identified by BBC method (Separate File).

Table S5. Lists of degraded proteins with cleavage sites during apoptosis (Separate File).

Table S6. List of the whole proteome on beads identified by LC-MS/MS (Separate File).

Table S7. The statistical representation analysis of 509 high confident substrates in biological process by Panther (Separate File).

A

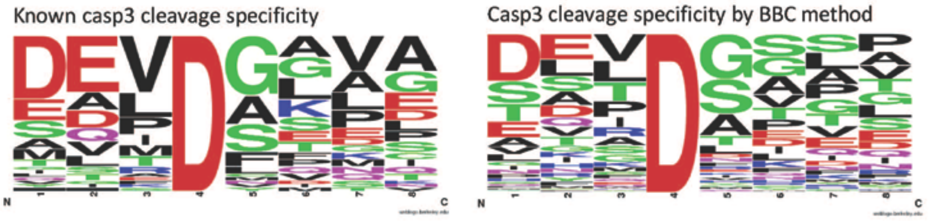

B

>sp|Q13177|PAK2_HUMAN Serine/threonine-protein kinase(PAK2)

MSDNGELEDK PPAPPVRMSS TIFSTGGKDP LSANHSLKPL PSVPEEKKPR
HKIISIFSGT EKGSKKKEKE RPEISPPSDF EHTIHVGFDA VTGEFTGMPE
QWARLLQTSN ITKLEQKKNP QAVLDVLKFY DSNTVKQKYL SFTPPEKDGF
PSGTPALNAK GTEAPAVVTE EEDDDEETAP PVIAPRPDHT KSIYTRSVID
PVPAPVGDSH VDGAAKSLDK QKKKTKMTDE EIMEKLRTIV SIGDPKKKYT
RYEKIGQGAS GTVFTATDVA LGQEVAIKQI NLQKQPKKEL IINEILVMKE
LKNPNIVNFL DSYLVGDELF VVMEYLAGGS LTDVVTETCM DEAQIAAVCR
ECLQALEFLH ANQVIHRDIK SDNVLLGMEG SVKLTDFGFC AQITPEQSKR
STMVGTPYWM APEVVTRKAY GPKVDIWSLG IMAIEMVEGE PPYLNENPLR
ALYLIATNGT PELQNPEKLS PIFRDFLNRC LEMDVEKRGS AKELLQHPFL
KLAKPLSSLT PLIMAAKEAM KSNR

>sp|O75934|SPF27_HUMAN Pre-mRNA-splicing factor SPF27(BCAS2)

MAGTGLVAGE VVVDALPYFD QGYEAPGVRE AAAALVEEET RRYRPTKNYL
SYLTAPDYSA FETDIMRNEF ERLAARQPIE LLSMKRYELP APSSGQKNDI
TAWQECVNNS MAQLEHQAVR IENLELMSQH GCNAWKVYNE NLVHMIEHAQ
KELQKLRKHI QDLNWQRKNM QLTAGSKLRE MESNWVSLVS KNYEIERTIV
QLENEIYQIK QQHGEANKEN IRQDF

Protein	Sequence#	Gene names	Ratio H/L	log(Ratio H/L)
Q13177	PPEKD.GFPSGTPALNAK.GTEAP	PAK2	5.853	3.285
Q13177	KLEQK.KNPQAVLDVLKF.YDSNT	PAK2	5.577	3.024
Q13177	SIYTR.SVIDPVPAPVGDSHVD.GAAKS	PAK2	35.852	5.712
Q13177	KKKTK.MTDEEIMEK.LRTIV	PAK2	24.965	5.440
Q13177	ALNAK.GTEAPAVVTEEEDDDEETAPPVIAPRPDHTK.SIYTR	PAK2	4.679	3.044
Q13177	STGGK.DPLSANHSLKPLPSVPEEK.KPRHK	PAK2	4.006	2.791
Q13177	ALNAK.GTEAPAVVTEEEDDDEETAPPVIAPR.PDHTK	PAK2	7.388	3.543
O75934	EVVVD.ALPYFDQGYEAPGVR.EAAAA	BCAS2	10.184	4.011
O75934	APGVR.EAAAALVEEETRR.YRPTK	BCAS2	3.893	2.752

In the sequence, "Red color" underlined represents the motif caspase-3 cleaved and "Blue color" represents the peptide identified by MS.
"#" indicates the sequence with four amino acids added before and after of the peptide sequence identified by MS.

Figure S1. Analysis of caspase-3 specificity. (A) WebLogo of the consensus motif from known caspase-3 substrates and putative substrates identified by BBC method. (B) Peptides identified from substrates PAK2 and BCAS2 by LC-MS/MS in the BBC method.
